# 1119. Assessment of Vancomycin Pharmacokinetic Parameters in Pediatric Patients After Liver Transplantation

**DOI:** 10.1093/ofid/ofab466.1312

**Published:** 2021-12-04

**Authors:** Ronaldo Morales, Vanessa D Juodinis, Daniela Carla de Souza, Silvia Regina C Jorge Santos

**Affiliations:** 1 São Paulo University, São Paulo, Sao Paulo, Brazil; 2 Sírio-Libanês Hospital, São Paulo, Sao Paulo, Brazil

## Abstract

**Background:**

Vancomycin is largely prescribed to treat gram-positive bacterial infections in pediatric patients after liver transplantation with the same empirical doses prescribed in other critical conditions due to the absence of pharmacokinetic studies in this population. The objective of this investigation was to describe the vancomycin pharmacokinetic parameters and to assess the vancomycin percentage of target attainment with empirical regimen.

**Methods:**

Prospective and longitudinal study with pediatric post-liver transplantation patients who received at least 48 hours of vancomycin between January 2020 and May 2021. Patients with acute or chronic renal failure or receiving renal replacement therapy were excluded. Vancomycin therapy started with 40-60mg/kg daily, one-hour infusion. The pharmacokinetic parameters were determined by one-compartment model with first-order kinetics using near steady-state postdistributional peak and trough within the same dosing interval. Therapeutic target was defined as vancomycin 24-hour area under the curve/minimum inhibitory concentration (AUC^ss^_0-24_/MIC) ≥ 400 and < 600. The study protocol was approved by the local ethics committee.

**Results:**

We included 18 sets of peak/trough serum concentrations obtained from 12 patients. The patients had median age of 11 (interquartile range [IQ] 8-16) months. The found vancomycin clearance, volume of distribution and half-life values were, respectively, 2.1 (IQ 1.4-2.8) mL/kg/min, 0.6 (IQ 0.5-0.7) L/kg and 3.2 (IQ 2.3-4.0) hours. After the initial dose regimen, 5 (42%) patients reached the therapeutic target.

**Conclusion:**

Using the one-compartment model, we evaluate the pharmacokinetic parameters of vancomycin in pediatric patients after liver transplantation. Most of patients did not reach the therapeutic target with empirical regimen, so it is prudent to monitor the exposure to vancomycin directly by AUC/MIC ratio to maximize antimicrobial efficacy.

**Disclosures:**

**All Authors**: No reported disclosures

